# *In Vivo* Evaluation of α7 Nicotinic Acetylcholine Receptor Agonists [^11^C]A-582941 and [^11^C]A-844606 in Mice and Conscious Monkeys

**DOI:** 10.1371/journal.pone.0008961

**Published:** 2010-02-01

**Authors:** Jun Toyohara, Kiichi Ishiwata, Muneyuki Sakata, Jin Wu, Shingo Nishiyama, Hideo Tsukada, Kenji Hashimoto

**Affiliations:** 1 Division of Clinical Neuroscience, Chiba University Center for Forensic Mental Health, Chiba, Japan; 2 Positron Medical Center, Tokyo Metropolitan Institute of Gerontology, Tokyo, Japan; 3 Central Research Laboratory, Hamamatsu Photonics K.K., Shizuoka, Japan; Vrije Universiteit Amsterdam, Netherlands

## Abstract

**Background:**

The α7 nicotinic acetylcholine receptors (nAChRs) play an important role in the pathophysiology of neuropsychiatric diseases such as schizophrenia and Alzheimer's disease. The goal of this study was to evaluate the two carbon-11-labeled α7 nAChR agonists [^11^C]A-582941 and [^11^C]A-844606 for their potential as novel positron emission tomography (PET) tracers.

**Methodology/Principal Findings:**

The two tracers were synthesized by methylation of the corresponding desmethyl precursors using [^11^C]methyl triflate. Effects of receptor blockade in mice were determined by coinjection of either tracer along with a carrier or an excess amount of a selective α7 nAChR agonist (SSR180711). Metabolic stability was investigated using radio-HPLC. Dynamic PET scans were performed in conscious monkeys with/without SSR180711-treatment. [^11^C]A-582941 and [^11^C]A-844606 showed high uptake in the mouse brain. Most radioactive compounds in the brain were detected as an unchanged form. However, regional selectivity and selective receptor blockade were not clearly observed for either compound in the mouse brain. On the other hand, the total distribution volume of [^11^C]A-582941 and [^11^C]A-844606 was high in the hippocampus and thalamus but low in the cerebellum in the conscious monkey brain, and reduced by pretreatment with SSR180711.

**Conclusions/Significance:**

A nonhuman primate study suggests that [^11^C]A-582941 and [^11^C]A-844606 would be potential PET ligands for imaging α7 nAChRs in the human brain.

## Introduction

The nicotinic acetylcholine receptors (nAChRs) are ligand-gated ion channels that are distributed throughout the human central nervous system (CNS) and that each consist of five subunits (a combination of α and β subunits). At present, nine α (α2–α10) and three β (β2–β4) subunits have been identified in humans [1]. Among the several nAChRs subtypes in the CNS, the homomeric α7 and heteromeric α4β2 subtypes are predominant in the brain [2]. These subtypes are best characterized in terms of their ligand selectivity, since they can be studied by means of binding techniques: [^3^H]cytisine or [^3^H]nicotine can label α4β2 nAChRs, and [^125^I]α-bungarotoxin or [^3^H]methyllycaconitine ([^3^H]MLA) is used to label α7 nAChRs [3]. In the mouse brain, high concentrations of α7 nAChRs are found in the pons, hippocampus and colliculi [4]. In the rat brain, high densities of α7 nAChRs are found in the hippocampus, hypothalamus, and cortical areas, whereas they are expressed to lesser degrees in the striatum and cerebellum [5]. Although the distribution of α7 nAChRs in primates is still not completely known, the available data suggest that it does not differ greatly, overall, from that in rodents. The α7 nAChRs are most dense in the thalamic nuclei and moderately dense in the hippocampus, prefrontal cortex, caudate, putamen, and substantia nigra in the monkey brain [6].

Several lines of evidence suggest that α7 nAChRs play a role in the pathophysiology of neuropsychiatric diseases such as schizophrenia, Alzheimer's disease, anxiety, depression, and drug addiction, and that α7 nAChRs are the attractive therapeutic targets for these diseases [2], [7]–[15]. Studies using postmortem human brain samples have demonstrated alterations in the levels of α7 nAChRs in the brains of patients with schizophrenia [16], [17] and Alzheimer's disease [18]–[20]. It is thus of great interest to examine whether α7 nAChRs are altered in the living brain of patients with neuropsychiatric diseases such as schizophrenia and Alzheimer's disease. It is also of interest to measure the receptor occupancy of potential therapeutic α7 nAChR drugs in the intact human brain. Therefore, several researchers have made an effort to develop radioligands that can be used to selectively and quantitatively examine the distribution of α7 nAChRs in the human brain with positron emission tomography (PET) and single photon emission computed tomography (SPECT). However, the development of such radioligands has been challenging due to a lack of lead structures with high affinity and with functional groups that can be labeled with PET and SPECT radioisotopes.

Recently, our group developed the 1,4-diazabicyclo-[3.2.2]nonane analog 4-[^11^C]methylphenyl 2,5-diazabicyclo[3.2.2]noane-2-carbocylate ([^11^C]CHIBA-1001) and confirmed its selective uptake in the conscious monkey brain by PET [21]. Until now, [^11^C]CHIBA-1001 has been the only PET ligand available for clinical trials employing α7 nAChR imaging in the human brain [22]. While [^11^C]CHIBA-1001 demonstrates some α7 nAChR-selective properties, we considered that it would be beneficial to identify another lead structure, and began a search for a more suitable PET radioligand for imaging α7 nAChRs in the human brain. Recently, a new series of octahydropyrrolo[3,4-*c*]pyrrole derivatives were described by Abbott Laboratories as ligands for the nAChRs [23], [24]. We chose two octahydropyrrolo[3,4-*c*]pyrrole derivatives as selective α7 nAChRs agonists for labeling with carbon-11, 2-methyl-5-[6-phenylpyridazine-3-yl]octahydropyrrolo[3,4-*c*]pyrrole (A-582941) and 2-(5-methyl-hexahydro-pyrrolo[3,4-c]pyrrol-2-yl)-xanthene-9-one (A-844606), since these compounds were previously reported to be potent and selective α7 nAChR agonists. A-582941 displaced specific binding of the α7 nAChR radioligand [^3^H]A-585539 to membranes of the rat brain and human frontal cortex with *K*_i_ values of 10.8 and 17 nM, respectively [25], [26]. In contrast, A-582941 was found to have much lower affinity for heteromeric α4β2 subtypes, as measured using [^3^H]cytisine binding to rat brain membranes (*K*_i_>100,000 nM) [26]. Also, A-582941 (10 µM) did not show significant displacement of binding of >75 binding sites, with the single exception being 5-HT_3_ receptors, in which it displaced [^3^H]-BRL 43694 (Granisetron) binding (>85% at 10 µM). The *K*_i_ value of A-582941 for 5-HT_3_ receptors was 154 nM, which was ∼15-fold higher than that for α7 nAChRs. A-844606 displaced [^3^H]MLA binding to the rat brain membrane with an IC_50_ value of 11 nM [27]. In contrast, A-844606 exhibited negligible displacement of [^3^H]cytisine binding to α4β2 nAChRs (IC_50_>30,000 nM) [27].

The goal of this study was to radiolabel the two potent and selective α7 nAChR agonists A-582941 and A-844606 with the positron emitter carbon-11, and to evaluate their potential for the *in vivo* imaging of α7 nAChRs in the human brain.

## Results

### Radiosynthesis

**Figure 1:**
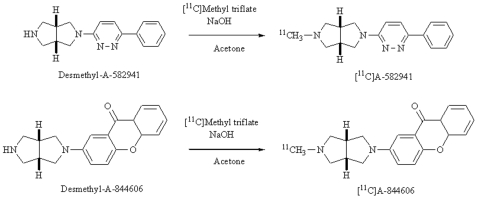
Radiosynthesis of [^11^C]A-582941 and [^11^C]A-844606.

Radiosynthesis of [^11^C]A-582941 and [^11^C]A-844606 by *N*-[^11^C]-methylation of the desmethyl precursor was carried out under various concentrations of NaOH as a base (Figure 1). The use of [^11^C]methyl triflate in acetone with two equimolar amounts of NaOH led to a sufficient radiochemical yield of each compound. The radiochemical yields using [^11^C]methyl triflate under these conditions for [^11^C]A-582941 and [^11^C]A-844606 were 16.8±11.98% (*n* = 8) and 40.0±16.8% (*n* = 7), respectively. Large excess or equimolar amounts of NaOH as a base led to a slight decrease of the radiochemical yields of [^11^C]A-582941 (10.3% for equimolar; 13.2% for 10 equimolar). The total preparation time for each tracer, including purification and formulation, was approximately 30 min from the end of irradiation. The radiochemical purities of each tracer were over 97%, and the specific activities at 30 min after the end of irradiation were in the range of 15–108 GBq/µmol for each radiotracer. The absence of any residual traces of the starting materials was verified by high performance liquid chromatography (HPLC) analysis.

### Tissue Distribution Study

The results of the tissue distribution studies of the two radiotracers in mice are summarized in Tables 1 and 2. The highest initial uptake (percentage of injected doses per gram of tissue: %ID/g) of [^11^C]A-582941 was found in the lungs followed by the kidneys, pancreas, heart, liver, small intestines, spleen, brain and muscle (Table 1). The radioactivity level of [^11^C]A-582941 was low in the blood. The level of radioactivity in the brain increased for the first 5 min and then gradually decreased. The highest initial uptake (%ID/g) of [^11^C]A-844606 was found in the lungs followed by the kidneys, heart, brain, pancreas, small intestines, muscle, liver and spleen (Table 2). The radioactivity levels of [^11^C]A-844606 were low in the blood. The level of radioactivity in the brain increased gradually, peaked at 30 min, and then decreased.

**Table 1:** Tissue distribution of radioactivity after intravenous injection of [^11^C]A-582941 into mice.

	% Injected dose/g tissue (mean ± S.D., *n* = 4)				
	1 min	5 min	15 min	30 min	60 min
Blood	1.84±0.43	0.89±0.04	0.76±0.06	0.64±0.06	0.57±0.05
Heart	4.33±0.82	2.16±0.13	1.72±0.17	1.39±0.10	1.02±0.08
Lung	18.09±4.18	12.52±8.59	7.02±0.10	6.06±0.31	4.13±0.15
Liver	3.59±0.45	10.52±0.69	12.38±1.68	9.87±0.80	7.24±0.74
Pancreas	4.65±0.76	7.47±0.54	5.80±0.80	4.68±0.51	3.69±0.42
Spleen	2.70±0.31	5.53±0.38	5.13±0.72	3.64±0.14	2.89±0.90
Kidney	13.87±1.58	8.77±0.79	6.02±0.97	4.83±0.77	3.74±0.20
S. intestine	3.13±0.51	4.49±0.60	4.61±0.60	5.33±1.20	4.53±1.21
Muscle	2.02±0.34	1.40±0.16	1.06±0.09	0.82±0.07	0.66±0.05
Brain	2.61±0.33	3.92±0.49	3.63±0.28	2.68±0.29	1.85±0.10

**Table 2:** Tissue distribution of radioactivity in mice after intravenous injection of [^11^C]A-844606.

	% Injected dose/g tissue (mean ± S.D., *n* = 4)				
	1 min	5 min	15 min	30 min	60 min
Blood	1.03±0.26	0.63±0.08	0.53±0.06	0.54±0.06	0.40±0.03
Heart	9.34±1.26	4.09±0.33	2.11±0.12	1.64±0.17	0.85±0.12
Lung	45.85±9.29	23.39±1.09	13.92±3.07	8.98±2.04	3.61±0.62
Liver	2.49±0.77	4.48±0.29	6.90±0.34	7.59±0.94	5.39±0.46
Pancreas	3.76±1.08	6.67±1.25	7.77±0.61	6.52±0.72	2.68±0.85
Spleen	1.75±0.65	4.84±0.71	7.34±0.17	5.82±1.29	2.47±0.40
Kidney	12.37±2.63	12.01±0.91	8.62±0.97	6.38±0.98	2.99±0.27
S. intestine	2.92±0.88	4.74±0.84	7.86±0.97	9.60±3.11	8.35±0.42
Muscle	2.53±0.61	1.93±0.40	1.35±0.20	1.06±0.16	0.55±0.06
Brain	4.16±0.85	6.26±0.97	7.31±1.16	7.96±0.62	4.27±0.49

To detect the specific binding for α7 nAChRs, a blocking experiment was carried out using the selective α7 nAChR agonists (SSR180711, unlabeled A-582941 or A-844606), and the selective α4β2 nAChR agonist A-85380 (Tables 3, 4, 5, and 6). In vehicle-treated mice, the hippocampal (α7 rich) uptake of [^11^C]A-582941 was not significantly higher than the cerebellar (α7 poor) uptake at 15 min after injection. In contrast, the hippocampal uptake of [^11^C]A-844606 in vehicle-treated mice was slightly higher than the cerebellar uptake at 30 min after injection, although the difference was not statistically significant. None of the three compounds (SSR180711, A-85380 or A-844606) decreased the uptake of [^11^C]A-582941 in brain tissues, with the exception of SSR180711 in the medulla oblongata (Table 3). Carrier-loading decreased the uptake of [^11^C]A-582941 in the medulla oblongata and midbrain (Table 4). In the case of [^11^C]A-844606, co-injection of the three compounds (SSR180711, A-85380 and A-582941) did not decrease the uptake of [^11^C]A-844606 in brain tissues (Table 5). In contrast, carrier-loading decreased the uptake of [^11^C]A-844606 in all the regions of the brain tissues (Table 6).

**Table 3:** Effects of co-injection of the subtype-selective nAChRs agonists on the brain tissue uptake of radioactivity 15 min after injection of [^11^C]A-582941 into mice.

	% Injected dose/g tissue (mean ± S.D., *n* = 5)			
	Control	A-85380	A-844606	SSR180711
Blood	0.88±0.10	0.85±0.06	0.91±0.10	1.06±0.09 **
Cerebellum	3.71±0.19	3.76±0.50	3.62±0.10	3.32±0.25
Medulla oblongata	4.23±0.08	3.99±0.49	4.04±0.19	3.15±0.14*
Hypothalamus	3.51±0.60	3.97±0.65	3.50±0.87	3.49±0.36
Hippocampus	4.05±0.52	3.92±0.54	4.20±0.55	4.63±0.51
Striatum	4.03±0.45	4.33±0.75	3.94±0.89	4.19±1.04
Midbrain	4.09±0.52	4.52±0.68	4.29±0.11	3.91±0.45
Cerebral cortex	4.59±0.25	4.53±0.61	4.78±0.19	5.02±0.46
Whole brain	4.23±0.23	4.26±0.57	4.27±0.18	4.21±0.39

Significant differences (p<0.05): *decrease and **increase compared to the control (ANOVA with Bonferroni's post-hoc tests).

The co-injected dose of each nAChRs agonist was 1 mg/kg.

**Table 4:** Effects of carrier-loading on the brain tissue uptake of radioactivity 15 min after injection of [^11^C]A-582941 into mice.

	% Injected dose/g tissue (mean ± S.D., *n* = 5)			
	Control	0.01 mg/kg	0.1 mg/kg	1.0 mg/kg
Blood	0.74±0.11	0.74±0.06	0.84±0.10	0.88±0.11
Cerebellum	2.73±0.21	2.62±0.16	2.76±0.48	2.33±0.38
Medulla oblongata	3.66±0.24	3.18±0.35	3.30±0.41	2.63±0.29*
Hypothalamus	3.10±0.38	2.87±0.56	3.15±0.31	2.79±0.33
Hippocampus	3.24±0.62	3.18±0.38	3.61±0.41	3.28±0.31
Striatum	3.36±0.38	3.30±0.25	3.61±0.27	3.45±0.19
Midbrain	3.70±0.29	3.02±0.41*	3.25±0.14	3.05±0.27*
Cerebral cortex	4.08±0.50	3.64±0.40	3.98±0.31	3.75±0.40
Whole brain	3.63±0.35	3.25±0.30	3.53±0.30	3.22±0.32

* Significant decrease (p<0.05) compared to the control (ANOVA with Bonferroni's post-hoc tests).

**Table 5:** Effects of co-injection of the subtype-selective nAChRs agonists on the brain tissue uptake of radioactivity 30 min after injection of [^11^C]A-844606 into mice.

	% Injected dose/g tissue (mean ± S.D., *n* = 5)			
	Control	A-85380	A-582941	SSR180711
Blood	0.57±0.11	0.54±0.12	0.61±0.07	0.52±0.06
Cerebellum	4.54±0.63	6.22±1.26	5.17±0.97	5.10±0.69
Medulla oblongata	5.25±0.95	5.46±1.15	5.58±1.26	5.35±0.33
Hypothalamus	4.21±1.55	5.00±0.96	4.76±1.17	4.74±0.70
Hippocampus	4.46±0.90	5.58±1.26	6.48±1.31	6.26±0.64
Striatum	5.01±2.00	6.47±1.38	6.81±1.05	7.32±1.84
Midbrain	5.43±0.75	6.84±1.71	5.80±1.46	6.69±0.79
Cerebral cortex	7.63±0.98	7.57±1.48	8.38±1.22	8.43±1.07
Whole brain	5.83±0.56	6.64±1.33	6.71±1.09	6.82±0.64

No significant differences (p<0.05) between the control and each drug-treated group (ANOVA with Bonferroni's post-hoc tests).

The co-injected dose of each nAChRs agonist was 1 mg/kg.

**Table 6:** Effects of carrier-loading on the brain tissue uptake of radioactivity 30 min after injection of [^11^C]A-844606 into mice.

	% Injected dose/g tissue (mean ± S.D., *n* = 5)			
	Control	0.01 mg/kg	0.1 mg/kg	1.0 mg/kg
Blood	0.38±0.06	0.28±0.08	0.33±0.02	0.26±0.04*
Cerebellum	6.19±0.58	4.76±1.09*	4.85±0.67	3.49±0.46*
Medulla oblongata	6.42±1.08	4.38±0.54*	4.59±0.48*	3.37±0.36*
Hypothalamus	6.39±2.01	3.97±0.89*	4.07±0.60*	3.75±0.44*
Hippocampus	7.66±2.58	4.57±0.40*	4.66±0.27*	4.14±0.75*
Striatum	7.43±2.04	4.54±0.79*	5.33±1.49	4.57±0.99*
Midbrain	7.71±1.29	4.98±0.57*	5.61±0.55*	3.98±0.61*
Cerebral cortex	10.08±0.57	7.02±1.25*	6.85±0.54*	5.22±0.72*
Whole brain	8.02±0.71	5.54±0.83*	5.73±0.34*	4.34±0.61*

* Significant decrease (p<0.05) compared to the control (ANOVA with Bonferroni's post-hoc tests).

### Metabolite Analysis

By deproteinization, most of the radioactivity of the plasma and brain tissues was recovered in the soluble fraction in the metabolite analysis of [^11^C]A-582941 (>95%) and [^11^C]A-844606 (>85%). HPLC analysis of the plasma showed two major labeled metabolites in addition to [^11^C]A-582941 (retention time = 6.4 min). The retention times of the two major metabolites were 2.4 and 4.3 min. In contrast, these metabolites were present at only negligible levels in the brain. At 15 min after injection of [^11^C]A-582941, the percentages of the unchanged form in the brain and plasma were 95.4±1.9% and 40.5±7.1% (*n* = 3), respectively, and the corresponding figures were 97.4±0.8 and 26.8±6.6% (*n* = 3) at 30 min after injection. Similarly, in the case of [^11^C]A-844606, two major labeled metabolites were found in the plasma (retention times = 2.0 and 4.0 min) in addition to [^11^C]A-844606 (retention time = 8.8 min). Again, these metabolites were present at only negligible levels in the brain. The percentages of the unchanged form in the brain and plasma were 95.9±1.8% and 39.6±3.4% (*n* = 3), respectively, at 15 min, and 94.9% (*n* = 2) and 28.9±5.1% (*n* = 3), respectively, at 30 min.

### Conscious Monkey PET Studies

**Figure 2:**
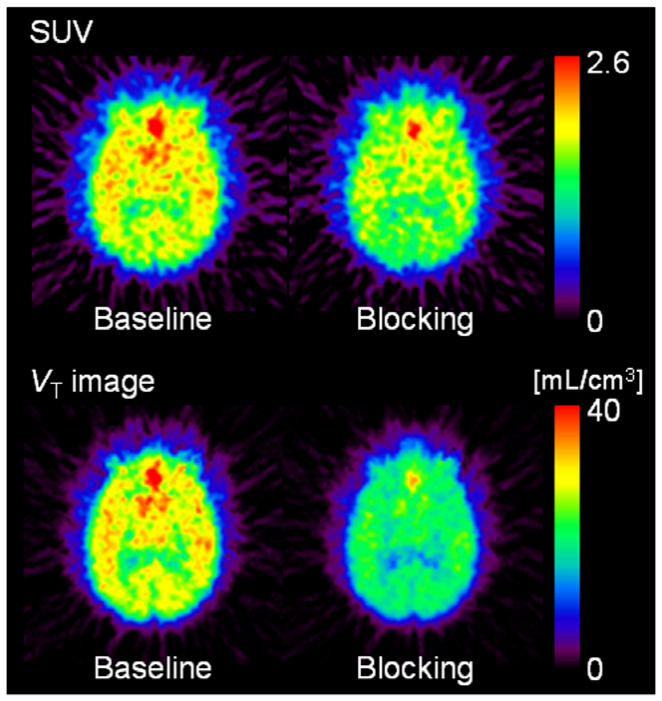
Baseline and SSR180711-blocking PET images of the monkey brain with [^11^C]A-582941. Upper: Static images acquired from 70 to 90 min after injection of [^11^C]A-582941, were expressed as a standardized uptake value (SUV). Lower: Parametric images for the total distribution volume of [^11^C]A-582941 were generated using Logan graphical analysis. SSR180711 (5 mg/kg) was intravenously injected into the monkey 30 min before the injection of [^11^C]A-582941.

**Figure 3:**
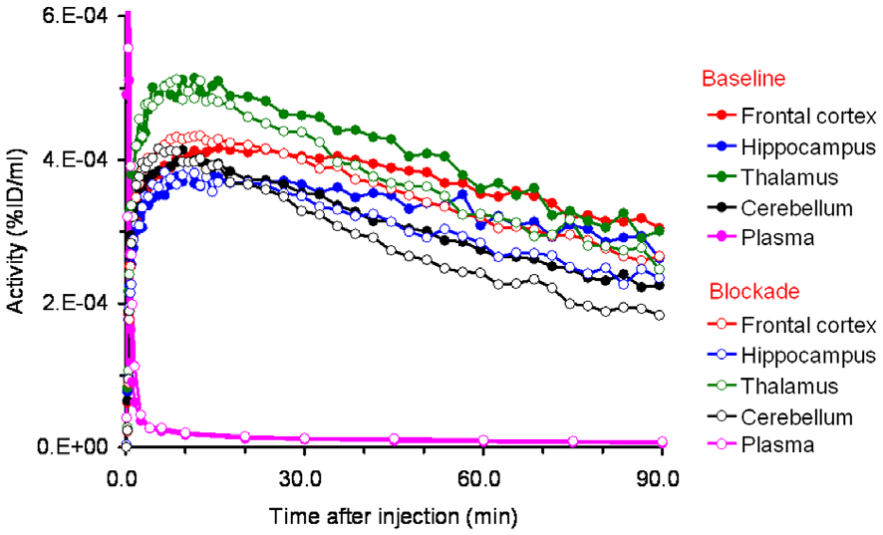
Time-activity curves of radioactivity in four brain regions (frontal cortex, thalamus, hippocampus, and cerebellum) and metabolite corrected plasma after intravenous injection of [^11^C]A-582941 in baseline and SSR180711-blocking PET scans. The monkey was given intravenously saline and SSR180711 (5.0 mg/kg, i.v.) in the baseline (*filled symbols*) and SSR180711-blocking (*open symbols*) scans, respectively, 30 min after injection of [^11^C]A-582941. Radioactivity was expressed as a percentage of injected doses per ml of tissue (%ID/ml).

Figure 2 shows the static and corresponding parametric total distribution volume (*V*_T_) images of [^11^C]A-582941 in the brain of a conscious monkey at baseline and under SSR180711-blocking conditions. Blocking effects by pretreatment with SSR180711 are seen in both images. Figure 3 shows their time-activity curves (TACs) in the four brain regions. In the baseline scan (*n* = 4), radioactivity in the four brain regions (the thalamus, frontal cortex, hippocampus, and cerebellum) peaked at 10 min. The highest accumulation of radioactivity was found in the thalamus, but the regional differences were small. Under the SSR180711-blocking condition, the radioactivity level peaked slightly earlier and decreased slightly more quickly.

The total and metabolite-corrected plasma radioactivity levels decreased rapidly (data not shown). Thin-layer chromatography (TLC) analysis revealed that the percentages of the unchanged form of [^11^C]A-582941 decreased rapidly at baseline (*n* = 4): 93.3±2.7%, 91.6±1.9%, 89.9±1.2%, 81.6±2.8%, 71.6±4.1%, 42.8±7.8%, 34.0±7.4%, 26.9±5.3%, 23.0±3.4%, and 19.2±2.6%; and under the SSR180711-blocking condition (*n* = 1): 93.2%, 93.9%, 93.0%, 74.2%, 56.7%, 28.3%, 23.2%, 20.4%, 22.2%, and 15.9% at 0.3, 0.7, 1.1, 6.0, 10, 30, 45, 60, 75, and 90 min, respectively.

In Logan graphical analysis, the rank order of *V*_T_ values (mean ± SD; range) was the thalamus (30.4±4.1; 24.6–34.0)> frontal cortex (29.2±4.7; 23.2–34.5)> hippocampus (28.0±3.8; 22.8–31.6)> cerebellum (23.0±3.4; 18.0–25.4). Pretreatment with SSR180711 significantly decreased *V*_T_ (∼30%) (Table 7).

**Table 7:** Total distribution volume (*V*_T_) of [^11^C]A-582941 and [^11^C]A-844606 in the baseline and SSR180711 pre-treatment conditions and receptor blocking rate by SSR180711 (*n* = 1).

	[^11^C]A-582941			[^11^C]A-844606		
	*V*_T_ (ml/g)			*V*_T_ (ml/g)		
	Baseline	SSR180711	% decrease*	Baseline	SSR180711	% decrease*
Cerebellum	25.4	17.3	32.0	95.9	52.8	45.0
Frontal cortex	34.5	23.0	33.4	132.2	65.3	50.6
Thalamus	34.0	23.6	30.5	141.5	69.3	51.0
Hippocampus	31.6	20.4	35.5	123.1	57.5	53.3

* *V*_T_ decrease was calculated as follow: [(*V*_T_ of baseline) – (*V*_T_ of SSR180711)]/(*V*_T_ of baseline)×100.

**Figure 4:**
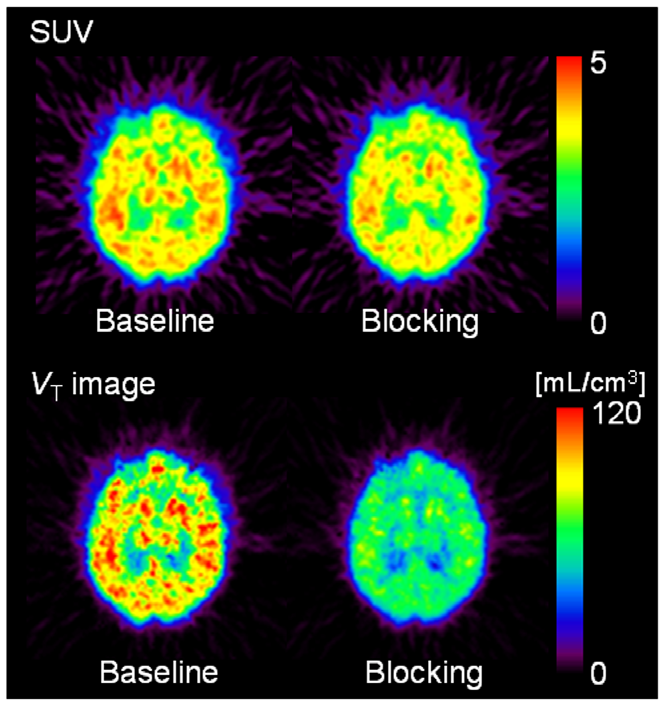
Baseline and SSR180711-blocking PET images of monkey brain with [^11^C]A-844606. Upper: Static images acquired from 70 to 90 min after injection of [^11^C]A-844606, were expressed as a standarized uptake value (SUV). Lower: Parametric image for the total distribution volume of [^11^C]A-844606 were generated using Logan graphical analysis. SSR180711 (5 mg/kg) was intravenously injected into the monkey 30 min before the injection of [^11^C]A-844606.

**Figure 5:**
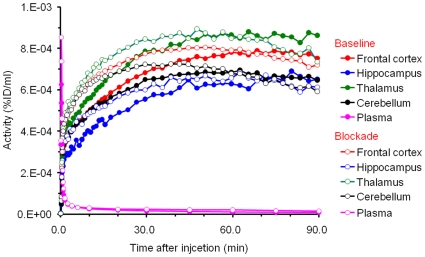
Time-activity curves of radioactivity in four brain regions (frontal cortex, thalamus, hippocampus, and cerebellum) and metabolite corrected plasma after intravenous injection of [^11^C]A-844606 in baseline and SSR180711-blocking PET scans. The monkey was given intravenously saline and SSR180711 (5.0 mg/kg, i.v.) in the baseline (*filled symbols*) and SSR180711-blocking (*open symbols*) scans, respectively, 30 min after injection of [^11^C]A-844606. Radioactivity was expressed as a percentage of injected doses per ml of tissue (%ID/ml).

Figures 4 and 5 show the static and *V*_T_ images of [^11^C]A-844606, and their TACs, respectively, in a monkey brain in baseline and SSR180711-blocking conditions. The blocking effect by pretreatment with SSR180711 was visualized much more clearly in the *V*_T_ images than in the static images. At baseline, the radioactivity in three of the four brain regions increased gradually over 90 min, with the exception being the cerebellum, where the radioactivity reached a plateau at 40 min. Under the SSR180711-blocking condition, the accumulation of radioactivity occurred slightly more quickly, reached a plateau at 40–60 min, and then decreased slightly. Both the total and metabolite-corrected plasma radioactivity decreased rapidly (data not shown).

The percentages of the unchanged form of [^11^C]A-844606 rapidly decreased in baseline (*n* = 3): 83.2±2.9%, 78.3±2.5%, 68.7±4.8%, 53.6±3.3%, 46.9±7.3%, 28.2±13.9%, 21.0±5.4%, 22.2±4.9%, 14.9±4.7%, and 13.4±4.2%; and under the SSR180711-blocking condition (*n* = 1): 90.7%, 87.1%, 80.4%, 63.3%, 55.2%, 28.7%, 24.0%, 16.8%, 13.2%, and 10.2% at 0.3, 0.7, 1.1, 6.0, 10, 30, 45, 60, 75, and 90 min, respectively.

The rank order of *V*_T_ values (mean ± SD; range, *n* = 3) was the thalamus (138.6±42.3; 95.0–179.4)> frontal cortex (130.9±40.4; 89.8–170.6)> hippocampus (118.5±33.6; 82.9–149.6)> cerebellum (95.2±32.8; 62.1–127.7). The *V*_T_ was significantly decreased (∼50%) by pretreatment with SSR180711 (Table 7).

## Discussion

In the present study, we successfully obtained the carbon-11-labeled selective α7 nAChR agonists A-582941 and A-844606, which possessed an octahydropyrrolo[3,4-*c*]pyrrole core structure. Methylation of *N*-, *O*-, or *S*-nucleophile with [^11^C]methyl triflate is the widely used tool for introducing a short-lived carbon-11 positron emitter into organic molecules. This strategy was successfully employed in the radiosynthesis of [^11^C]A-582941 and [^11^C]A-844606 by reaction of the corresponding desmethyl-precursors with [^11^C]methyl triflate. The amount of the base was optimized at two equivalent moles of the corresponding precursor. We also investigated its *in vivo* target specificity in mice and conscious monkeys.

Previous *in vitro* membrane-binding and pharmacological studies showed the high affinity and selectivity of A-582941 and A-844606 for α7 nAChRs [27], [28]. The target affinity of A-582941 (*K*_i_ = 10.8 nM) and A-844606 (IC_50_ = 11 nM) was high in comparison to the CHIBA-1001 (IC_50_ = 45.8 nM; *K*_i_ = 35 nM), of which a carbon-11-labeled analog has been used for imaging of α7 nAChRs in a clinical study [21]. The *K*_i_ values for CHIBA-1001 were calculated by the method of Cheng and Prusoff [29]. For calculation of the *K*_i_ value for CHIBA-1001, a value of 1.67 nM was used as the K_D_ for α-bungarotoxin. These results encouraged us to evaluate the possibility of using [^11^C]A-582941 and A-844606 for the *in vivo* imaging of α7 nAChRs in the brain.

In the *in vivo* distribution study in mice, both tracers showed high brain uptake. [^11^C]A-844606 showed higher brain uptake than [^11^C]A-582941. The hippocampal uptake of [^11^C]A-582941 was not significantly higher than the cerebellar uptake at 15 min after injection. In contrast, the hippocampal uptake of [^11^C]A-844606 was slightly higher than cerebellar uptake but not significant at 30 min after injection. These findings indicate the high nonspecific binding of [^11^C]A-582941 and a few receptor-specific binding of [^11^C]A-844606 in the mouse brain, because α7 nAChRs density is high in the hippocampus and low in the cerebellum in mice [4]. This high level of nonspecific binding of [^11^C]A-582941 was also confirmed in the competitive drug treatment studies. Co-injection with the selective α7 nAChR agonists SSR180711 and A-844606 did not decrease the regional brain uptake of [^11^C]A-582941. In the carrier-loading studies, a significant decrease of radioactivity was observed only in the medulla oblongata. These data imply that a limited fraction of the [^11^C]A-582941 taken up into the mouse brain was involved in receptor-specific binding *in vivo*.

In the competitive drug treatment studies, co-injection with the selective α7 nAChR agonists SSR180711 and A-582941 also could not decrease the regional brain uptake of [^11^C]A-844606. On the other hand, the carrier-loading studies indicated a significant decrease in uptake in both the hippocampus (target tissue) and cerebellum (non-target tissue). These data suggest that saturable binding sites of [^11^C]A-844606 are present in the mouse brain but may be different from α7 nAChRs *in vivo*.

However, it is well known that *in vivo* evaluations of specific binding in mice have some limitations and sometimes lead to false-negative results. First, the uptake studies employing dissected tissue are usually performed at a single time-point that is reasonably selected based on tissue time-activity curve (tTAC) obtained in the tissue distribution study. The kinetic analysis of the PET study of the monkey brain indicated that the specific binding was evaluated from overall data of the PET scan: 93 min scan in the present study. Indeed, in our current study, the specific binding of the tracers in mice was evaluated at the time of highest brain uptake under the baseline condition. Second, in the *in vivo* evaluations of the specific binding of tracers, pretreatment with blockers is usually more effective than co-injection of the same blockers. In the present studies using mice, the cold-displacement and blocking experiments were performed using the co-injection method. Indeed, an additional, unknown effect might have been produced by co-injection of selective α7 nAChR agonists (A-582941 and SSR180711) at a dose of 1 mg/kg, which resulted in enhanced [^11^C]A-844606 uptake in the brain tissues. When looking at the tTACs in the monkey brain, an enhanced uptake of [^11^C]A-844606 was also observed for approximately 60 min after the injection. Similar phenomena have sometimes been observed during the development of *in vivo* radioligands [30]–[32]. Moreover, we previously reported that [^11^C]doxepin was representative of a radioligand engendering these phenomena [33]. Notwithstanding the clinical usefulness of [^11^C]doxepin PET for measurement of the histamine H_1_ receptor occupancy rates of antihistamine agents [34], the specific binding of [^11^C]doxepin to histamine H_1_ receptors is very low in rodents, and is not detected in guinea pigs. However, kinetic analysis of the results of a PET study of [^11^C]doxepin showed 30% specific binding in the monkey brain, and a larger specific binding rate (36%) in the human brain [33]. As observed in the [^11^C]doxepin study using rodents, our negative results in mice are hardly expected from the *in vitro* studies cause the target affinity of A-582941 and A-844606 are higher than that of CHIBA-1001. Theoretically, kinetic analysis of PET study results in the monkey brain is far preferable to an uptake study using co-injection at a single time-point in rodents. We therefore consider that more suitable evaluations were performed by the monkey PET studies.

An *in vivo* PET study using conscious monkeys demonstrated a high accumulation [^11^C]A-582941 and [^11^C]A-844606 in the brain. In contrast to the mouse brain, the regional distribution of both tracers in the monkey brain is consistent with the distribution of α7 nAChRs. In rhesus monkeys, α7 nAChRs are distributed at the highest level in the thalamus, at a moderate level in the cortex, and at a low level in the cerebellum [35]–[38]. Figures 3 and 5 demonstrate a high uptake of both tracers in the thalamus and cortical regions and a low uptake in the cerebellum. However, the regional differences were small and the average *V*_T_ ratio of the α7 nAChR-rich thalamus to the α7 nAChR-poor cerebellum was below <2.0. Distinct from the mice data, the uptake of both tracers in the monkey brain regions was blocked by pretreatment with the selective α7 nAChR agonist SSR180711. Because the 1,4-diazabicyclo-[3.2.2]nonane skeleton of SSR180711 is different from the 3,7-diazabicyclo[3.3.0]octane skeleton of A-582941 and A-844606, this data might indicate the selectivity of [^11^C]A-582941 and [^11^C]A-844606 for α7 nAChRs. However, these positive characteristics, which indicate the specific binding of the tracer to α7 nAChRs, were diminished by the fact that these compounds also showed the same levels of blocking in the cerebellum. On the other hand, the possibility that the uptake of these tracers was due to the existence of α7 nAChRs in the cerebellum has not been ruled out. In fact, a human postmortem brain study has shown that [^125^I]α-bungarotoxin binding in the cerebellum is at the same level as that of the cortex [39], [40]. In the monkey brain, the α7 nAChR mRNA expression pattern resembles that of the postmortem human brain [40], [41].

Quantitative analysis lead out the significant *V*_T_ reduction in the brain regions of these tracers pretreated with SSR180711. However, the inter-subject differences of *V*_T_ in the baseline scan of these tracers were massive, and this difference was at the same level as the *V*_T_ reduction in the blocking study. *V*_T_ is the ratio at equilibrium of the total tissue concentration to the metabolite-corrected plasma concentration. Therefore, not only the changes of tTAC but also the changes of the plasma time activity curve (pTAC) will affect the *V*_T_. Indeed, the integral of pTAC of these tracers was obviously increased with SSR180711 pretreatment (data not shown), and might have caused the significant *V*_T_ reduction in the brain regions of these tracers.

Nevertheless, as mentioned above, the 30–50% *V*_T_ reduction rate was larger than that of [^11^C]doxepin (30%) in the monkey PET study. Thus, even though a high nonspecific binding of these tracers in the experimental animals are still exists, these compounds have chance to proceed to the preliminarily clinical evaluation when the clinical safety of these tracers are confirmed.

A species difference in the peripheral metabolism of [^11^C]A-582941 was found between mice and monkeys. The finding that [^11^C]A-582941 was more stable peripherally in monkeys than in mice is a good property for a PET ligand in monkeys: the percentages of the intact form in the plasma of mice and monkeys were 26.8% and 42.8%, respectively, at 30 min post-injection. In contrast, a species difference between mice and monkeys was not found in the peripheral metabolism of [^11^C]A-844606. The percentages of the intact form in the plasma of mice and monkeys were 28.9% and 28.2%, respectively, at 30 min post-injection.

A-582941 exhibits favorable physical properties for a CNS-active drug, with a low molecular weight (280 Da for the free base) and moderate lipophilicity (clog P = 2.3) [28]. The molecule possesses two basic sites, the *N*-methylated tertiary amine and aminopyridazine, with pK_a_ values of 8.75 and 4.44, respectively. At physiological pH, the molecule exists substantially in the monoprotonated state, and the log D at pH 7.4 is estimated to be 1.0. A-844606 also possesses one basic site, the *N*-methylated tertiary amine. Perhaps ion-pair interactions between the basic nitrogen and the charged acidic head groups of phospholipids membranes induce nonspecific binding. Although we cannot identify the cause of the species difference between mice and monkeys, some structural characteristics might be responsible for the high nonspecific binding in the mouse brain.

In general, a radioligand for *in vivo* imaging of neuronal receptors should possess the following properties: 1) a high signal-to-noise ratio that is linked to high binding affinity and low nonspecific binding, 2) good blood-brain barrier penetration and rapid clearance from the blood, 3) appropriate kinetics, 4) high receptor selectivity, 5) good radiation dosimetry/toxicology properties, and 6) appropriate radiochemistry. From previous *in vitro* and pharmacological studies of A-582941 and A-844606, we confirmed the 4) high receptor selectivity. In this study, we also confirmed that these compounds showed 2) good blood-brain barrier penetration and rapid clearance from the blood, and 6) appropriate radiochemistry. Also, the regional brain uptake of these compounds reached equilibrium with an ^11^C imaging time window. However, our results also showed that improvements and further considerations of categories 1) and 5) criteria are still needed. Although most studies of the distribution of α7 nAChRs in the brain are qualitative, one study using [^125^I]MLA showed a maximum number of binding sites of 68±3 fmol/mg proteins in the rat cerebral cortex and another, using [^3^H]MLA, demonstrated 59±4 fmol/mg in the mouse hippocampus [4], [41]. These α7 nAChR concentrations suggest that ligands with low nanomolar affinities should be successful for imaging α7 nAChRs *in vivo*. Indeed, with a *K*_d_ of 10 nM, comparable to A-582941 and A-844606, and a B_max_ of 70 fmol/mg (∼7 nM), the B_max_/*K*_d_ value is calculated at <1. This also indicates that further structural modification of octahydropyrrolo[3,4-*c*]pyrrole analogs toward high binding affinity may provide suitable imaging agents for α7 nAChRs. Very recently, Bunnelle *et al.* [42] reported that the replacement of the terminal phenyl of A-582941 by an indolyl group resulted in a potent and selective α7 ligand (*K*_i_ = 0.24 nM; >400,000-fold selective vs. α4β2-subtype). However, as stated by Ding *et al.* [43], we have to keep in mind that the high *in vitro* affinity of a ligand does not guarantee its suitability as an *in vivo* ligand. The poor ability to predict the behavior of chemical compounds *in vivo* based on log P values and affinities emphasizes the need for more knowledge in this area.

The specific activities of α7 nAChRs radioligands is an important factor to be considered. First, a radioligand of low mass is required so as not to saturate the binding sites. Second, use of radiotracers with more highly specific activity is more likely to ensure that the radiolabeled compound does not elicit any pharmacological effects when administered. Therefore, an ideal *in vivo* radiotracer for the α7 nAChRs should be an antagonist. However, a radiolabeled antagonist for α7 nAChRs with sufficiently high affinity for *in vivo* imaging has yet to be identified.

In conclusion, although an inter-species difference in the distribution of [^11^C]A-582941 and [^11^C]A-844606 was observed between rodents and non-human primates, our non-human primate study of [^11^C]A-582941 and [^11^C]A-844606 will present a helpful leads to finding the novel useful PET ligands for imaging α7 nAChRs in the human brain. Our results also showed that octahydropyrrolo[3,4-*c*]pyrrole may be a lead structure with high affinity and with functional groups that can be labeled with PET isotopes.

## Materials and Methods

### General

The reference compounds (A-582941 and A-844606) and their desmethyl precursors (desmethyl-A-582941 and desmethyl-A-84606) were synthesized according to the method described previously [23], [24], [28]. SSR180711 was also synthesized by a previously described method [21]. All other chemical reagents were obtained from commercial sources. Male ddY mice were obtained from Tokyo Laboratory Animals Co., Ltd (Tokyo, Japan). The animal studies were approved by the institutional ethics committees for animal experiments at the Tokyo Metropolitan Institute of Gerontology and Chiba University. The PET study with monkeys was approved by the institutional ethics committees for animal experiments at the Hamamatsu Photonics K.K. and Chiba University. The PET study with monkeys was performed at the Central Research Laboratory of Hamamatsu Photonics K.K., Hamamatsu, Japan, in accordance with recommendations of the U.S. National Institutes of Health and the guidelines of the Central Research Laboratory, Hamamatsu Photonics K.K. Monkeys were monitored closely and animal welfare is assessed on a daily basis, and if necessary several times a day. This includes veterinary examinations to make sure animals are not suffering. If animals experience pain they receive pain medications. If pain can not be relieved, or if veterinary examination reveals signs of suffering that cannot be relieved by analgesics, antiemetics, or antibiotic therapy, animals are euthanized.

#### 2-[^11^C]Methyl-5-[6-phenylpyridazine-3-yl]octahydropyrrolo[3,4-c]pyrrole ([^11^C]-A-582941)

[^11^C]A-582941 was synthesized by [^11^C]methylation of desmethyl-A-582941 with [^11^C]methyl triflate prepared using an automated synthesis system as previously described (Figure 1) [44]. [^11^C]Methyl triflate was trapped in acetone (0.25 ml) containing 0.25 mg (1 µmol) of desmethyl-A-582941 and 10 µl (2 µmol) of 0.2 M aqueous NaOH as a base. The reaction was carried out at room temperature for 1 min. After 1.3 ml of CH_3_CN/50 mM CH_3_COONH_4_ (20/80, v/v) had been added, the reaction mixture was applied to HPLC using a reverse phase column (YMC-Pack ODS-A: 10 mm inner diameter ×250 mm length; YMC Co., Ltd., Kyoto, Japan) together with a UV absorbance detector (260 nm) and a semiconductor radiation detector. The mobile phase was a mixture of CH_3_CN and 50 mM CH_3_COONH_4_ (20/80, v/v) at a flow rate of 6 ml/min. The [^11^C]A-582941 fraction (retention time: 15.7 min for [^11^C]A-582941 and 7.7 min for desmethyl-A-582941) was corrected and evaporated to dryness. The residue was dissolved in physiological saline and filtered through a 0.22 µm membrane. The labeled compound was analyzed by HPLC using a TSKgel Super-ODS column (4.6 mm inner diameter ×100 mm length; Tosoh Co., Ltd., Tokyo, Japan); the mobile phase was CH_3_CN/50 mM CH_3_COONH_4_ (35/65, v/v), the flow rate was 1.0 ml/min, and the retention time was 5.1 min for [^11^C]A-582941.

#### 2-(5-[^11^C]Methyl-hexahydro-pyrrolo[3,4-c]pyrrol-2-yl)-xanthene-9-one ([^11^C]A-844606)

[^11^C]A-844606 was prepared by a method similar to that for [^11^C]A-582941 with a slight modification in HPLC separation—namely, desmethyl-A-844606 (0.25 mg, 1 µmol) was used as the labeling precursor (Figure 1). After 1.3 ml of CH_3_CN/50 mM CH_3_COONH_4_ (35/65, v/v) had been added, the reaction mixture was applied to HPLC using the same reversed phase column. The mobile phase was a mixture of CH_3_CN and 50 mM CH_3_COONH_4_ (35/65, v/v) at a flow rate of 6 ml/min and the retention times were 11.8 min for [^11^C]A-844606 and 6.3 min for desmethyl-A-844606. Then the [^11^C]A-844606 fraction prepared for injection was analyzed by the same HPLC with a different mobile phase consisting of CH_3_CN/H_2_O/triethylamine (65/35/0.1, v/v) and a retention time of 7.0 min for [^11^C]A-844606.

### Tissue Distribution of the Tracers in Mice

Each tracer was intravenously injected into mice, and then the animals were killed by cervical dislocation at 1, 5, 15, 30 and 60 min after injection (*n* = 4). The body weight of the mice was 34.7±1.2 g. The injected doses of tracers were 2 MBq/0.049–0.051 nmol. Blood was collected by heart puncture and tissues were harvested. The carbon-11 in the samples was counted with an auto-gamma-counter (LKB Wallac Compu-gamma 1282CS, Turku, Finland) and the tissues were weighed. The tissue uptake of carbon-11 was expressed as the %ID/g.

In the other group of mice, a blocking experiment to determine the α7 nAChRs-specific regional brain uptake of the tracers was carried out. Each tracer (2 MBq/0.055–0.096 nmol) was co-injected with each of the following blockers into mice, and 15 ([^11^C]A-582941) or 30 ([^11^C]A-844606) min later the mice were killed (*n* = 5) and the tissue uptake of ^11^C was expressed as the %ID/g. The co-injected blockers were unlabeled A-582941 and A-844606, SSR180711 (α7n AChRs selective agonist, α7 nAChRs, IC_50_ = 30 and 18 nM for rat and human receptors of brain homogenates, respectively; α4β2, IC_50_>50,000 nM for human receptor) [45] or A-85380 (α4β2 nAChRs selective agonist, α7 nAChRs, *K*_i_ = 100 and 148 nM for rat and human receptors of brain homogenates, respectively; α4β2, *K*_i_ = 0.05 and 0.04 nM for rat and human receptors of brain homogenates, respectively) [46]. Each blocker was dissolved in 1N HCl to 40 mg/ml and diluted up to 0.4 mg/ml with saline. The co-injected dose was 1 mg/kg. In the control mice the same amount of 0.01 N HCl in saline was co-injected.

To investigate the carrier-loading effect of the tracer uptake in the mouse brain, each tracer (2 MBq/0.053–0.179 nmol) was co-injected with unlabeled A-582941 or A-844606. The co-injected doses were 0.01, 0.1 and 1 mg/kg. In the control mice, the same amount of 0.01 N HCl in saline was co-injected. Fifteen ([^11^C]A-582941) or 30 ([^11^C]A-844606) min later the mice were killed (*n* = 5) and the tissue uptake of ^11^C was expressed as the %ID/g.

### Metabolite Analysis

Each tracer (180 MBq/3.0–13.4 nmol) was intravenously injected into mice, and 15 and 30 min later the animals were killed by cervical dislocation (*n* = 3). Blood was removed by heart puncture using a heparinized syringe, and the brain was removed. The blood was centrifuged at 7,000×*g* for 1 min at 4°C to obtain plasma, which was denatured with 5 volume of CH_3_CN. The mixture was centrifuged under the same conditions, and the precipitate was re-suspended in 0.5 ml of CH_3_CN, followed by centrifugation. This procedure was repeated three times. The cerebral cortex (approximately 200 mg) was homogenized in 1.0 ml of 50% CH_3_CN. The homogenate was then treated as described above. The combined supernatant was diluted with 2 volumes of water and then analyzed by HPLC with a radioactivity detector (FLO-ONE 150TR; Packard Instrument, Meriden, CT). A Radial-Pak C18 column equipped with an RCM 8×10 module (8 mm ×100 mm; Waters, Milford, MA) was used with a mixture of CH_3_CN/H_2_O/triethyl amine (40/60/0.1 for [^11^C]A-582941 and 70/30/0.1 for [^11^C]A-844606, v/v/v) as the mobile phase at a flow rate of 2 ml/min. The elution profile was monitored with a radioactivity detector. The retention times of [^11^C]A-582941 and [^11^C]A-844606 were 6.4 and 8.8 min, respectively. The recovery in the eluate of the injected radioactivity was essentially quantitative.

### PET Study in Conscious Monkeys

Four young-adult male rhesus monkeys (*Macaca mulatta*) weighing from 4 to 6 kg were used for PET measurements. The monkeys were trained for the protocol as described previously [21]. The magnetic resonance images (MRI) of all monkeys were obtained with a Toshiba MRT-50A/II (0.5T) under anesthesia with pentobarbital. The stereotactic coordinates of PET and MRI were adjusted based on the orbitomeatal (OM) line with monkeys secured in a specially designed head holder [47]. At least 1 month before the PET study, an acrylic plate, with which the monkey was fixed to the monkey chair, was attached to the head under pentobarbital anesthesia as described previously [48].

PET data were collected on a high-resolution PET scanner (SHR-7700; Hamamatsu Photonics K.K., Hamamatsu, Japan). The camera consists of 16 detector rings and acquires 20 slices at a center-to-center interval of 3.6 mm with a transaxial resolution of 2.6 mm full width at half maximum [49]. After an overnight fast, the monkey was fixed to the monkey chair with stereotactic coordinates aligned parallel to the OM line. A cannula was implanted in the posterior tibial vein, and [^11^C]A-582941 or [^11^C]A-844606 (1152–1336 MBq/26.8–63.1 nmol) was injected into the monkey through the venous cannula. PET images were acquired over 91 min (10 sec ×6 frames, 30 sec ×6 frames, 1 min ×12 frames, and 3 min ×25 frames). PET scans were reconstructed using a filtered backprojection method in a 100×100 matrix, with a voxel size of 1.2 mm ×1.2 mm ×3.6 mm. Summed image of late phase (from 70 to 91 min post-injection) was calculated as a static SUV (activity/ml tissue)/(injected activity/g body weight) image. Each MRI was coregistered to an early (from 0 to 20 min) summed image using normalized mutual information, and ROIs were placed on the summed image with reference to the coregistered MRI. Three monkeys underwent [^11^C]A-582941 scan morning, and then two of the three monkeys underwent [^11^C]A-844606 afternoon (baseline). Within 6 months the last one of the three monkeys underwent two [^11^C]A-844606 scans in the baseline (morning) and blocking (afternoon) conditions 30 min after injection of SSR180711 (5.0 mg/kg, i.v.), and the fourth monkey underwent two [^11^C]A-582941 scans in the baseline and blocking conditions in the same way. Due to the very short half-life of ^11^C (20.4 min), a time lag of at least 3 hours between the two PET scans in an individual monkey in the same day provided a sufficient decay time of radioactivity (approximately 1/400 of the injected dose). Therefore, the level of radioactivity associated with the previous injection of labeled compound did not interfere with the next scan, as previously reported [50], [51].

For the semi-quantitative analysis of PET data, arterial samples were obtained every 8 sec from injection to 64 sec, and then again at 1.5, 2.5, 4, 6, 10, 20, 30, 45, 60, and 90 min after each tracer injection. Blood samples were centrifuged to separate the plasma, weighed, and subjected to radioactivity measurement. For metabolite analysis, methanol was added to some plasma samples, the resulting solutions were centrifuged, and the supernatants were developed with TLC plates (AL SIL G/UV; Whatman, Kent, UK) using a mobile phase of dichloromethane/diethyl ether/ethanol/triethylamine (20/20/2/2, v/v/v/v). At each sampling time point, the ratio of radioactivity in the unmetabolized fraction to that in the total plasma (metabolite plus unmetabolite) was determined using a phosphoimaging plate (BAS-1500 MAC; Fuji Film Co., Tokyo, Japan). The pTACs corrected for metabolite were obtained. The tTACs in each region of interest (ROI) in the brain were calculated as the %ID/ml or as a standardized uptake value (SUV), (activity/ml tissue)/(injected activity/g body weight). Using the tTACs and the metabolite-corrected pTAC, the *V*_T_ for each tracer was evaluated by Logan graphical analysis [52].

### Statistical Analysis

A one-way analysis of variance (ANOVA) with Bonferroni's post-hoc tests was used in comparing treated groups to controls. Differences with a p value<0.05 were considered to be statistically significant.
